# Clean or Pasteurised Milk

**Published:** 1908-03-21

**Authors:** F. Willington Lane

**Affiliations:** Old Palace House, Theobalds, Waltham Cross, Herts.


					Editors Letter-Box.
[Our correspondents are reminded that prolixity is a great
publication, and that brevity of style and conciseness of state?1
greatly facilitate early insertion. Ill
" CLEAN OR PASTEURISED MILK?"
Sir,?I am writing to call your attention to what app^
to me to be a contradiction in your issue of the 7th 1?..
for on the first page you give an article on " Clean ?
or Pasteurised Milk," in which you run down ste*1" ,
and pasteurised milk, speaking of them as undesirabl0 i
unnecessary. Then in the same issue, page 607, you #
an article on " Public Health and Hygiene," describe ?
the authority of Professor Matthew Hay, Medical Officer ?
Health for Aberdeen, a very serious outbreak of tyP ^
fever in Peterhead during 1907, which he traoed, ^ ^
doubt, to infection conveyed in the milk supply. ^
milk been sterilised instead of being consume'd in i^s
state, the outbreak would not have occurred. Surely ^ ^
shows that sterilising and pasteurising are desirable .
very necessary, and, in fact, the only means of prod?fl
a safe milk supply.
Yours truly.
,0)
I
F. WELLINGTON S'
Old Palace House, Theobalds, Waltham
Cross, Herts.
March 10, 1908. *
' ------ -> , -00^
. ? . The arguments in favour of clean milk in preifir ^
to pasteurised milk are not weakened in the least W 1
occurrence of outbreaks of fever due to unclean
taminated milk. Such outbreaks merely emphasis6 f 0
importance of an absolutely clean milk supply. Dr. ^ ^ F
might on similar grounds advocate the sterilisation ? ??
water used for drinking purposes and the washing of ei
utensils in preference to strict supervision of the
of supply.?Ed., The Hospital.

				

